# Drug repurposing for Chagas disease: *In vitro* assessment of nimesulide against *Trypanosoma cruzi* and insights on its mechanisms of action

**DOI:** 10.1371/journal.pone.0258292

**Published:** 2021-10-22

**Authors:** Joana D’Arc S. Trindade, Célio Geraldo Freire-de-Lima, Suzana Côrte-Real, Debora Decote-Ricardo, Marco Edilson Freire de Lima

**Affiliations:** 1 Instituto de Química, Departamento de Química Orgânica, Universidade Federal Rural do Rio de Janeiro, Seropédica, Rio de Janeiro, Brazil; 2 Instituto de Biofísica Carlos Chagas Filho, Universidade Federal do Rio de Janeiro, Ilha do Fundão, Rio de Janeiro, Brazil; 3 Instituto Oswaldo Cruz/Fiocruz, Laboratório de Biologia Estrutural, Rio de Janeiro, Brazil; 4 Instituto de Veterinária, Departamento de Microbiologia e Imunologia Veterinária, Universidade Federal Rural do Rio de Janeiro, Seropédica, Rio de Janeiro, Brazil; King Abdulaziz University, Faculty of Pharmacy, SAUDI ARABIA

## Abstract

Chagas disease is a neglected illness caused by *Trypanosoma cruzi* and its treatment is done only with two drugs, nifurtimox and benznidazole. However, both drugs are ineffective in the chronic phase, in addition to causing serious side effects. This context of therapeutic limitation justifies the continuous research for alternative drugs. Here, we study the *in vitro* trypanocidal effects of the non-steroidal anti-inflammatory drug nimesulide, a molecule that has in its chemical structure a toxicophoric nitroaromatic group (NO_2_). The set of results obtained in this work highlights the potential for repurposing nimesulide in the treatment of this disease that affects millions of people around the world.

## Introduction

**Fig 1:**
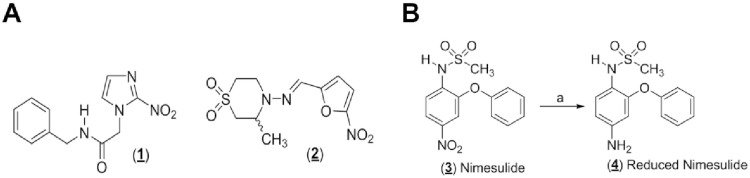
**A**. Chemical structures of benznidazole (BZN, **1**) and nifurtimox (NFX, **2**), the only drugs available to the treatment of Chagas disease. **B**. Synthesis of the aniline **4** through chemoselective reduction of nimesulide **3**. Reaction conditions: (a) Fe^0^, NH_4_Cl, ethanol/water, reflux (64%).

Chagas disease is one of the 17 neglected tropical diseases (NTDs) listed by the World Health Organization, with 6 to 7 million people infected worldwide,^1^ being related to underdevelopment and poverty [1]. However, in recent decades the spread of this parasitic infection has been reported to other regions due to climate changes, such as some countries in Europe and also in the United States, where different species of insect vectors infected by *T*. *cruzi* were found [2, 3]. The treatment of the disease in its acute phase is made only by two drugs benznidazole (BZN) (**1)** and nifurtimox (NFX) (**2**) (Fig 1A). These drugs have several side effects such as anorexia, weight loss, psychic changes, and others [4]. These therapeutic limitations to treat the infected patients point to the need to develop new alternatives for the treatment of this infectious disease. The growing understanding of the pathophysiology of Chagas disease, coupled with important international collaborations involving academia, industry and governments, may culminate in the development of more effective chemotherapeutic agents, with the potential to remove Chagas disease from the classification of a neglected tropical disease, mainly bringing new hope for the treatment of chronic chagasic patients. [5].

The discovery of a new drugs is an important achievement and traverses a long conventional path of research, from the identification of the molecular target to its registration and manufacture. The process can last from 10 to 17 years with a high cost involved [6]. Another equally relevant strategy shortens this time by implying new studies for an already known drug. Successful research results in its reuse for a different therapeutic action, proving to be more advantageous in terms of cost as well. Both approaches are important for identifying new trypanocidal compounds. However, finding a treatment faster and at a lower cost for neglected parasitic diseases is a differential that should not be overlooked. Currently, the repositioning approach has been facilitated by the availability of extensive drug library and by advances in genomics and bioinformatics [6].

Studies on nitroheterocycles drugs have advanced and may lead to an understanding of the mechanisms of antiparasitic action of these compounds [7]. It is well known that Non-Steroidal Anti-Inflammatory Agents (NSAIDs), such as nimesulide (NIM) (**3**, Fig 1B), are inhibitors of eicosanoids biosynthesis, lipid mediators with an important role in the mechanism of *T*. *cruzi* infection [8]. Our group had already characterized the modulation of COX-2 as a potential biochemical target for Chagas chemotherapy [9]. The fact that both nimesulide and benzonidazole have a nitroaromatic group, known to be toxophoric to *T*. *cruzi*, is another relevant point that stimulated us in this study. It is not the first time nimesulide has been evaluated for another therapeutic application other than anti-inflammatory. This drug has already been tested, for example, against the growth of cancer cells [10, 11] and as anti-diabetic [12]. From the data presented, we have understood that the study of the toxic activity of nimesulide against *T*. *cruzi* could shed light on the potential application of this anti-inflammatory drug in the treatment of chagasic patients. Recent reports have highlighted the dangers of co-infection of chagasic patients with SARS-CoV-2, due to the heart being one the most affected organs in patients with COVID-19 [13]. The fact that nimesulide is an anti-inflammatory drug makes this study even more relevant, since one of the problems associated with the chronic phase of Chagas disease is the development of myocarditis, which can lead to heart failure [14].

A widely used analysis technique for identifying cell damage caused by chemical agents is transmission electron microscopy (TEM). In this study, we sought to assess the ultrastructural effects on parasites after treatment with nitroaromatic nimesulide, comparing with the information available in the literature.

Although the ultrastructural effects caused on the parasite’s organelles by drug treatments are useful in the investigation of possible targets and possible mechanisms of action of these potential chemotherapeutic compounds [15–17] the literature states that with respect to *T*. *cruzi* there is still insufficient data on the exact path of cell death caused by different molecular agents, with evidence of death due to multiple factors. For example, changes in chromatin organization, presence of cytosolic myelin figures and formation of autophagosomal bodies, suggest an autophagic death process. However, it is possible to form bubbles in the membrane and changes in chromatin, indicating a possible apoptotic path. Other times, there is a rupture of the plasma membrane, which is related to death by necrosis. In other words, a variety of alterations can be observed, leading to the occurrence of more than one death path, when *T*. *cruzi* cells are challenged by different drugs [17, 18].

## Material and methods

### Chemistry

The equipment used in the characterizations and determination of purity grades of compounds can be found in S1 Text.

#### Extraction of nimesulide (3) from commercially available medicines

Nimesulide was extracted from tablets of medicines obtained on the market. The acid-base extraction was carried out according to the methodology described by Gonsalves et al. [19]. The white solid obtained was recrystallized twice from ethanol furnishing 0.9 g (90% yield) of highly pure nimesulide (**3**) [20, 21].

#### The chemoselective reduction of the nitro group of nimesulide

As shown in Fig 1B, to evaluate the role of nitroaromatic moiety of nimesulide structure, the aniline (**4**) prepared from nimesulide was obtained as described by Maia et al. [22], using metallic iron in presence of aqueous ammonium chloride and ethanol reagent. The aniline (**4**) (199 mg; 65% yield) was obtained as a white crystalline solid [21, 23].

The two products obtained were fully characterized through their melting points and spectra data (NMR ^1^H, ^13^C, and mass spectra) which are in full agreement with data previously described in the literature [21–23]. All data used in the characterization and determination of the degree of purity of the compounds (**3**) and (**4**) can be found in S1–S6 **Figs**; S1 and S2 **Tables**.

For the preparation of the solutions of nimesulide, reduced nimesulide and benznidazole, a stock solution of 30 mM of each was prepared using DMSO. The final concentration of drugs in each well was adjusted to 35 μM. The final concentration of DMSO in was 0.12%.

### Biological assessments

#### Animals and ethics statement

BALB/c mice of both sexes, aged 6–8 weeks, were obtained from the Veterinary Institute of the Federal Rural University of Rio de Janeiro, and kept in micro-isolators with food and water *ad libitum*. This study was carried out in strict accordance with the recommendations of the Guide for the Care and Use of Laboratory Animals, from the National Institutes of Health (United States). The protocol was approved by the Animal Experimentation Ethics Committee of the Veterinary Institute of the Federal Rural University of Rio de Janeiro (CEUA-IV, Authorization Number: 8819010217) and every effort was made to minimize animal suffering. All animal work was performed in accordance with Animal Research: Reporting of In Vivo Experiments (ARRIVE) guidelines and regulations.

#### Parasites

Trypomastigote forms from the blood of mice infected with *Trypanosome cruzi* (Y strain) were kindly provided by Dr. Mirian Cláudia de Souza Pereira, head of the Cellular Ultrastructure Laboratory (Oswaldo Cruz Foundation-Rio de Janeiro, RJ, Brazil), and were used to obtain the different evolutive stages of the parasite for the tests performed [24].

The metacyclic trypomastigotes of *T*. *cruzi* were obtained from cultures of LLCMK2 cells. After growth, the culture medium was discharged, and the cells were washed with phosphate-saline buffer (PBS). Then, the cells were trypsinized by incubation for 5 to 10 minutes at 37°C with a solution containing 1 mM ethylene diamino tetraacetic acid (Merck KGaA, Darmstadt, Germany) and 0.25% trypsin (Life Technologies) in PBS. The incubation was interrupted by the addition of PBS supplemented with 10% SFB, followed by centrifugation at 1500 rpm, for 6 minutes, at 20°C. The cells were resuspended in dilution from 1:5 to 1:10 in complete RPMI medium (Merck KGaA, Darmstadt, Germany). When the culture reached about 50% confluence, LLCMK2 cells were infected with the trypomastigotes of the *T*. *cruzi*, Y strain in a ratio of 5 to 10 parasites *per* cell. After 24 hours, non-internalized parasites were removed. The trypomastigotes used were obtained from the collection of LLCMK2 cell supernatant, on the 7^th^ and 9^th^ days after infection. LLCMK2 adherent epithelial cell lines were maintained in complete RPMI 1640 (Merck KGaA, Darmstadt, Germany) medium (2 mM glutamine, 50 mM β-mercaptoethanol, 100 mM pyruvate, 50 μg.mL^-1^ of gentamicin, non-essential amino acids, supplemented with 2.5% SFB), at 37°C with a humid atmosphere containing 5% CO_2_. All experiments were conducted with biosafety level 2.

#### Epimastigote growth

The epimastigotes of *T*. *cruzi* were grown in cardiac and brain tissue infusion medium (BHI, BD Bioscience) supplemented with 10% fetal bovine serum (SFB, Gibco), 20 μg.mL^-1^ of folic acid (Merck KGaA, Darmstadt, Germany), 12 μg.mL^-1^ of hemin (Merck KGaA, Darmstadt, Germany), 10 μg.mL^-1^ of gentamicin (Merck KGaA, Darmstadt, Germany), this medium being called complete BHI. The parasite cultures were kept in a BOD incubator at 27°C, passing every 7 days, using inoculum of 10^6^ parasites.mL^-1^ in 25 mL of complete BHI medium. The parasites were centrifuged at 2200 rpm for 10 minutes at 4°C, resuspended in 10 mL of complete BHI and quantified in a Neubauer chamber with 0.01% trypan blue (Riedel-de Haën, North Carolina, USA). The epimastigote forms used in the tests were fifth pass at most.

#### Peritoneal macrophages

Macrophages were obtained from BALB/c mice by washing the peritoneal cavity with 5 mL of DMEM (Gibco-Invitrogen) supplemented and without serum. For duplicate assays, cells were taken from the peritoneal lavage of 6 animals. The cells were counted in a Neubauer chamber using trypan blue (Merck KGaA, Darmstadt, Germany). Cell concentration was adjusted to 2x10^5^ cells.mL^-1^ in supplemented DMEM medium with 10% SFB. Then, 1 mL of this suspension was distributed in triplicates in microplates of 24 and 48 wells for cell culture (TPP^®^). The plates were incubated at 37°C under 5% CO_2_, for 3 hours.

#### Cytotoxicity assays

Cell toxicity was assessed using the XTT colorimetric method [25, 26]. The test was performed in the presence of the PMS electron acceptor. The peritoneal macrophages of BALB/c mice were cultured in 96-well microplates, with complete DMEM culture medium (10^5^ cells *per* well), maintained under the same experimental conditions as a control of the technique. The macrophages were then incubated with nimesulide at concentrations of 17 and 35 μM for 24 and 48 hours. After incubation, 50 μL of XTT solution (1.8 mg of XTT, 75 μL of PMS and 1.425 μL of PBS) were added to each well. After 48 h the reading was performed in a microplate reader equipped with a 490 nm filter (Spectramax M3).

#### Viability of *T*. *cruzi* epimastigotes treated with different concentrations of nimesulide

*T*. *cruzi* epimastigotes in exponential growth phase (96 h) were resuspended in complete BHI medium at the final concentration of 2.0 x 10^5^ parasites.mL^-1^. In 96-well culture microplates, 100 μL of the parasite culture were added and were treated or not with different concentrations of nimesulide ranging from 100 to 1.56 μM. The plates were incubated at 27°C for 72 hours in BOD. The MTT reduction assay was performed in order to analyze the viability of the parasites in each treatment [27]. The trypanocidal effect was assessed by the concentration of nimesulide required for 50% inhibition of epimastigotes growth (IC_50_) when compared to untreated control. Benznidazole was used as positive control.

#### Infection of macrophages with trypomastigotes

During the parasitic peaks, supernatant from infected LLCMK2 cells was centrifuged to obtain *T*. *cruzi* trypomastigotes. Then, the parasites were resuspended in complete RPMI medium, reaching a concentration of 1.0 × 10^7^ parasites.mL^-1^. The macrophages were infected in the ratio of 3 trypomastigotes per cell. The culture was at 37°C with a humid atmosphere containing 5% CO_2_. After 24 hours the wells were washed with PBS and maintained in complete DMEM, in the presence or not of nimesulide and 35 μM benznidazole and kept at 37°C with a humid atmosphere containing 5% CO_2_ for ten days. The parasites growth was evaluated after seven and nine days in culture, by counting in Neubauer’s chamber the free trypomastigote released.

#### Assessment of parasites viability by flow cytometry

Epimastigotes of *T*. *cruzi* (1.0 x 10^6^ parasites.mL^-1^) were incubated with 35 μM nimesulide for 24 hours. After treatment, they were centrifuged and resuspended in 1000 μL of buffer. The suspension was centrifuged again and then treated with annexin V-FITC (AV) and propidium iodide (IP) for analysis in flow cytometer (FACSCALIBUR Becton & Dickinson).

#### Ultrastructural analysis

Epimastigotes of *T*. *cruzi* (2 x 10^5^ parasites.mL^-1^) of third passage, in the exponential growth phase, were treated or not with 35 μM nimesulide for 24 hours and fixed with 2.5% glutaraldehyde, diluted in. 0.1 M sodium cacodylate, containing 3.5% sucrose. The medium was kept under pH 7.2 and 4°C for 1 hour. Then, the parasites were washed in the same buffer and post-fixed with 1% osmium tetroxide diluted in 0.1 M sodium cacodylate buffer at pH 7.2 and 4°C for 1 h. Then, the cells were washed in the same buffer, dehydrated in acetone gradient (30%, 50%, 70%, 90% and 100%) and incorporated in PolyBed 812 resin. After polymerization, ultrafine sections were obtained in Leica ULTRACUT S UCT (Leica Microsystems’ Instruments, Wetzlar, Germany) contrasted with 5% uranyl acetate and, afterwards, with 1% lead citrate. The analysis was performed using a transmission electron microscope (Jeol JEM-1011; Tokyo, Japan) at the Rudolf Barth Electron Microscopy Platform (Fiocruz-Rio de Janeiro, Brazil).

#### Statistical analysis

To assess the effect of nimesulide on the growth of parasites the data were analysed by the GraphPad Prism version 6 program, in one-way ANOVA analysis tests for independent samples, followed by Dunnet’s “t” test. The significance values were represented in the graphs by (*) for values with p <0.05; (**) for values with p <0.01 and (***) for values with p <0.001.

## Results

### Chemistry

The purity grade of nimesulide extracted from the commercial medicine (≥98%) was determined by RP-HPLC (S7 Fig).

As shown in Fig 1B, in order to evaluate the role of nitroaromatic moiety of nimesulide structure, the aniline **4** was synthesized through a chemoselective reduction of the nimesulide’s nitro group in presence of metallic iron in mild acidic medium (aqueous NH_4_Cl) [22, 23]. This methodology furnished the amine **4** without touching other groups present in drug structure, such as the sulfonylanilide moiety. The reduction product had its degree of purity (≥ 98%) evaluated by HPLC (S8 Fig) previously to the biological evaluations.

### Biological assessments

#### Characterization of the cytotoxic effect of nimesulide under experimental conditions

**Fig 2:**
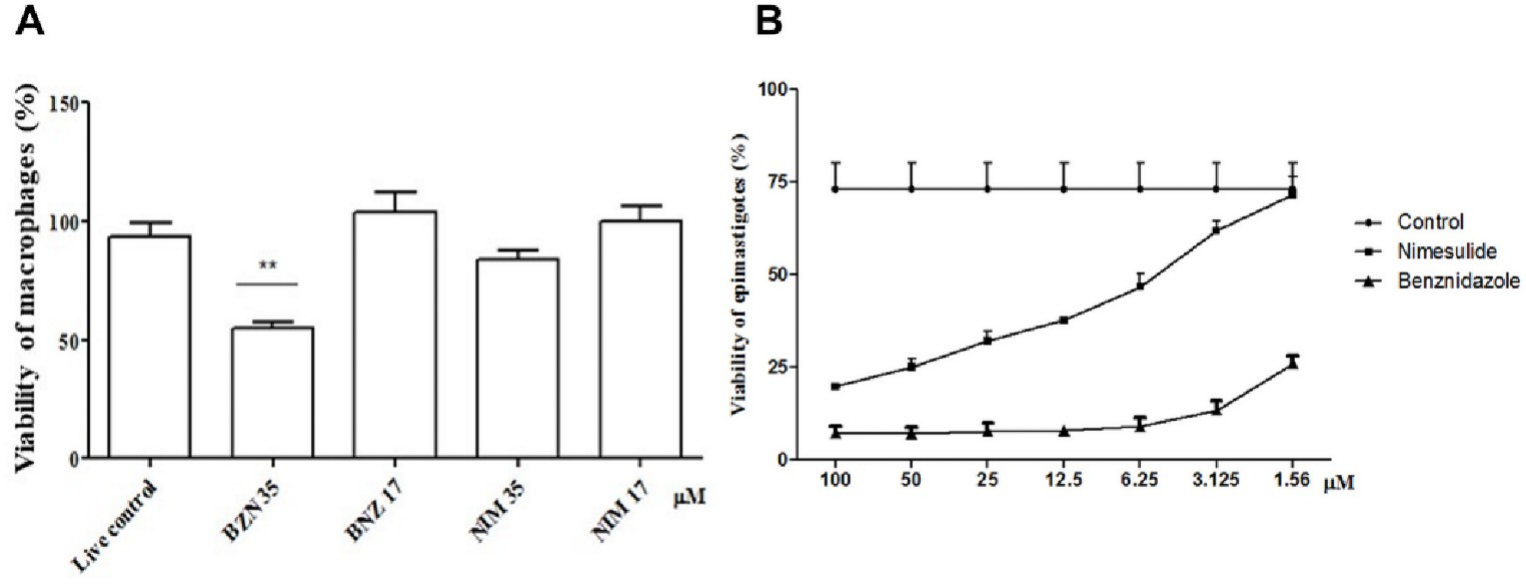
**A**. Cytotoxicity assay. Peritoneal macrophages of BALB/c mice incubated with benznidazole and nimesulide, at concentrations of 17 and 35 μM. Representative results from two independent experiments. **B**. Cell viability of *T*. *cruzi* epimastigotes (Y strain) in different concentrations of NIM and BNZ. Treatment after 48 hours of incubation at 27°C. In this assay, the inhibitory concentration (IC_50_) of nimesulide against epimastigotes was 12.93 ± 0.03 μM, and for benznidazole 3.82 ± 0.65 μM. Both results were for two independent experiments. Asterisks represent the significant difference from the untreated control (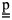
<0.05).

The experiment was conducted with the aim to infer whether the concentrations of nimesulide used in the treatments of epimastigotes would impair mammalian cell viability. The assay was conducted by incubating peritoneal macrophages of BALB/c mice with two concentrations of nimesulide, under the same experimental conditions used to epimastigotes. The results obtained showed that nimesulide does not have any toxic effects against murine macrophages, even at the highest concentration evaluated. nimesulide was less toxic to murine macrophages than benzonidazole, as shown in Fig 2A.

#### Cell viability of *T*. *cruzi* (Y strain) epimastigotes treated with nimesulide

After treatment of epimastigotes with nimesulide (**3**), in concentrations ranging from 2.0 to 100 μM, cell death was observed in a dose-dependent manner, allowing the calculation of the inhibitory concentration for 50% of the epimastigote forms (IC_50_ = 12.93 ± 0.03 μM). No toxic effects on the parasites were observed at concentrations of less than 1 micromolar. In the same conditions benznidazole (used as control drug) showed IC_50_ = 3.82 ± 0.65 μM. However, when epimastigotes were treated in the same conditions with the chemically reduced aniline (**4**) no cell death was observed (IC_50_> 200 μM). A control assay with DMSO (solvent used in the dilutions) was done under the same conditions, showing no toxicity in the concentrations used. The results are shown in Fig 2B.

#### Nimesulide reduces *T*. *cruzi* replication in macrophages

**Fig 3:**
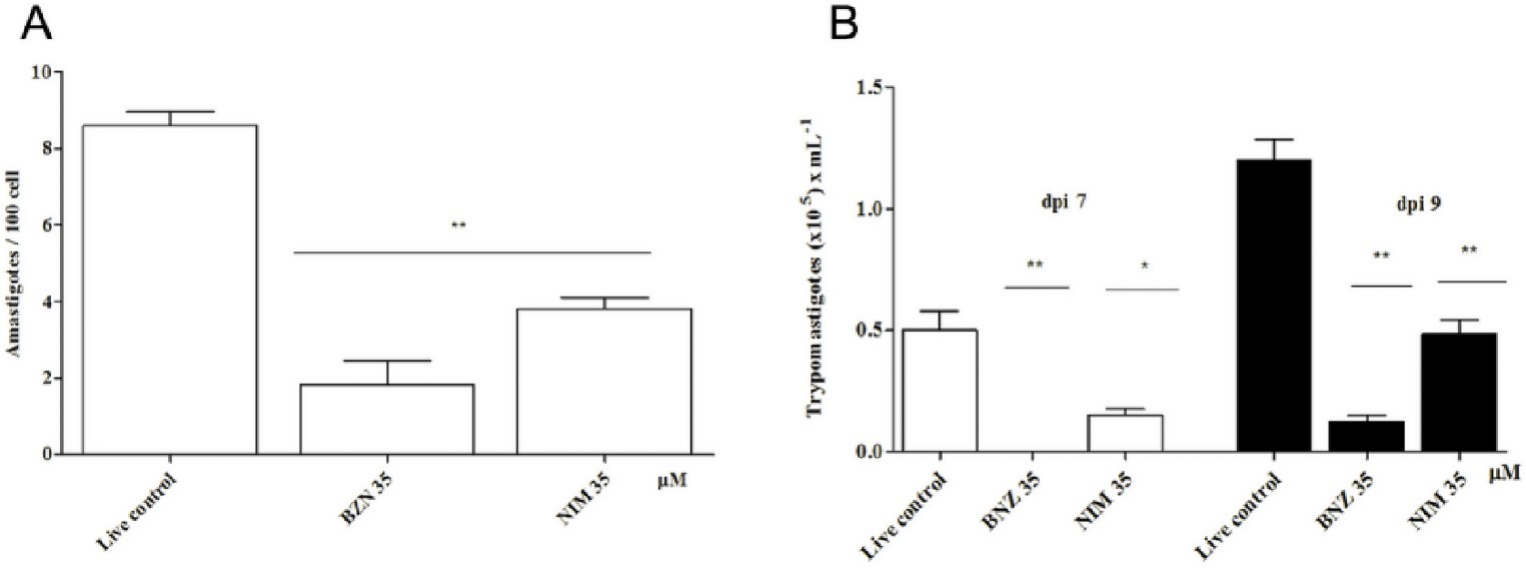
Infection of peritoneal macrophages in BALB/c mice with trypomastigotes, followed by treatment with benznidazole and nimesulide. **A**: Inhibition of amastigotes after 3 days of treatment. **B**: Release of trypomastigotes after 7 and 9 days of treatment. Representative results from two independent experiments. Both results were for two independent experiments. Asterisks represent the significant difference from the untreated control (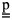
<0.05).

The treatment of peritoneal macrophages from BALB/c mice infected with *T*. *cruzi* trypomastigotes with nimesulide resulted in inhibition of intracellular amastigote replication, as shown in Fig 3A. It was observed a reduction in the number of viable trypomastigotes in the supernatant in the days 7 and 9 after infection, according to Fig 3B.

#### Assessment of parasites viability by flow cytometry

**Fig 4:**
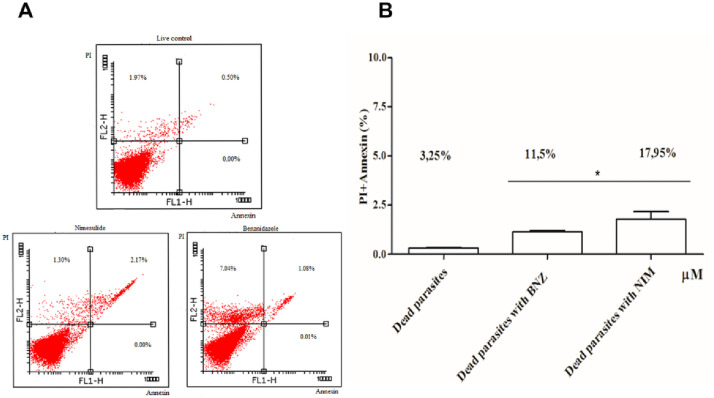
Elucidation of the type of cell death of epimastigotes of *T*. *cruzi* (Y strain), in the exponential growth phase, treated with nimesulide and benznidazole, at concentration of 35 μM, for 24 hours. **A**: The representative histograms of the parasites treated and marked with propidium iodide for necrosis death, in the FL2-H channel, and marked with annexin for apoptosis death, in the FL1-H channel. It is observed in the upper right region that nimesulide caused twice the death due to apoptosis and necrosis, simultaneously, compared to benznidazole. **B**. Graphical representation of the percentage of cell death in the cell culture treated with nimesulide. Control corresponds to viable AV-IP-cells. The effect of nimesulide was compared to the results obtained in the treatment with benznidazole and corresponds to late apoptotic cells AV+IP, for both treatments. Representative results from two independent experiments. Asterisk represent the significant difference from the untreated control (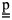
<0.05).

The analysis of parasites treated for 24 hours with nimesulide revealed staining for both probes, IP and AV. The results confirmed that treatment was like that of benznidazole, under the same conditions (Fig 4A and 4B).

#### Ultrastructural assessment of the effects of the drug nimesulide on *T*. *cruzi* epimastigotes in transmission electron microscopy

**Fig 5:**
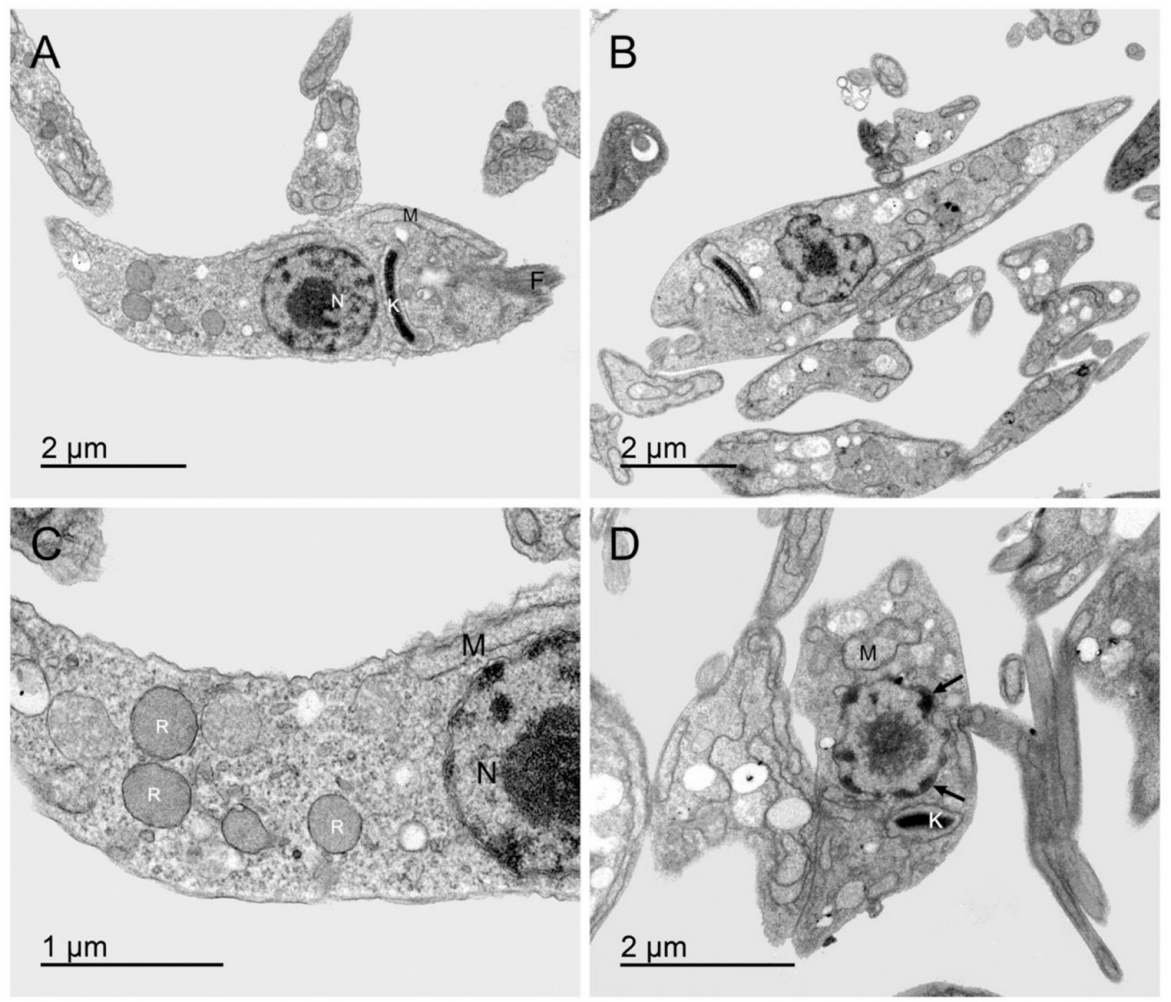
Transmission electron micrographs showing the ultrastructure of epimastigotes. **A**: Overview of control untreated. **B**: Overview of treatment with nimesulide for 24 h. **C**: Reservosomes (R), nucleus (N) and mitochondria (M) preserved in untreated control. **D**: Nucleus (N) with condensed heterochromatin located in sections of the nuclear envelope with a clear morphological change of this organelle (straight arrows), mitochondria (M) with altered morphology, showing very electrodense kinetoplast (K), after incubation with nimesulide.

In order to observe whether the treatment with nimesulide caused ultrastructural effects on the parasites, epimastigotes forms (Y strain) were treated or not with 35 μM nimesulide for 24 h. After the incubation, processing for transmission electron microscopy was performed and the material analysed indicated significant changes in cell organelles. The epimastigote cells in axenic culture revealed a characteristic organization of this evolutive form (Fig 5A). However, a close view of the parasites treated with nimesulide (**3**) indicated generalized changes (Fig 5B). Futhermore, untreated parasites showed intact mitochondria containing the kinetoplast, nucleus and preserved reservosomes (Fig 5C). Although, in the treated forms, the mitochondria appear with changes in its structure, quite dilated and without apparent ridges, containing the electron-dense, well-compacted kDNA, with exacerbated condensation, indicating a possible alteration in the architecture of the genetic material (Fig 5D).

**Fig 6:**
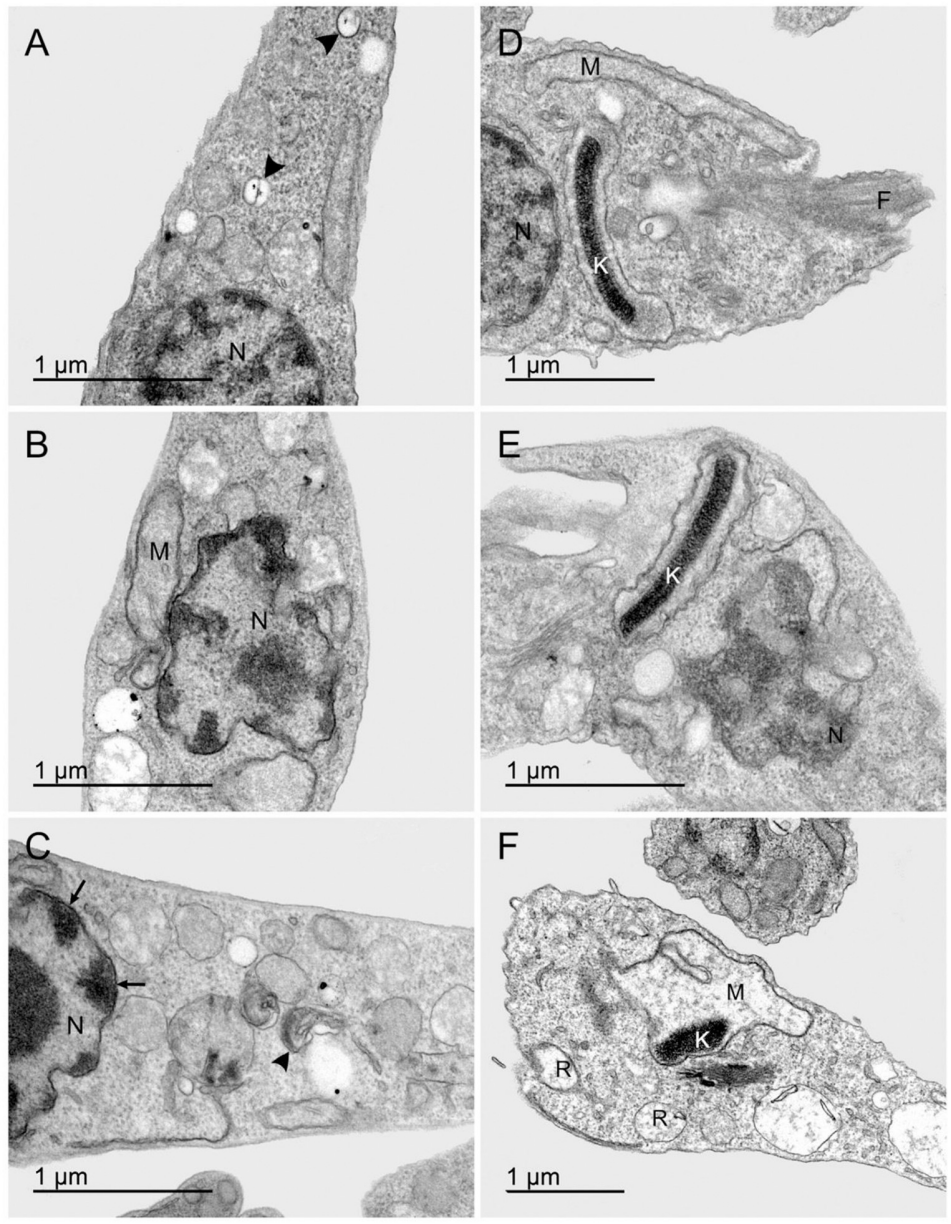
Effects of nimesulide on *T*. *cruzi*. **A**: Calciosomal acids distributed in the cytoplasm, indicated by arrowheads and intact nucleus (N), in the untreated control. After treatment with nimesulide, **B**: Nucleus (N) with disruption of the nuclear envelope, dilated mitochondria (M) and presence of vacuoles, and **C**: Disorganized nucleus (N) with heterochromatin adhered to some sections of the nuclear envelope (straight arrows), figures of myelin are present in the cytosol, represented by the arrowhead. **D**: Well-preserved mitochondrial profile, close to the plasma membrane, the kinetoplast (K) also shows the kDNA preserved in the mitochondrial portion between the nucleus (N) and the flagellum (F), in the untreated control. After treatment with nimesulide, **E**: The treatment induced profound alterations on nuclear morphology (N) and kinetoplast condensation (K), and **F**: Mitochondria (M) well dilated and with loss of ridges throughout the parasite body, electrodense kinetoplast (K) and presence of the reservosomes (R) with low content.

In addition, it was possible to highlight the presence of acidocalcisome in untreated parasites (Fig 6A), but treated parasites shows sections of the nuclear envelope adhered heterochromatin and cytosol it was possible to identify myelin figures (Fig 6B and 6C).

In general, the micrographs of the untreated parasites showed a characteristic mitochondrial profile, close to the plasma membrane, kinetoplast with kDNA preserved in the mitochondrial portion between the nucleus and the flagellum (Fig 6D). On the other hand, after incubation with nimesulide, mitochondria were well dilated, with loss of crests throughout the body of the parasites, electron-dense kinetoplast and presence of reservosomes with low content (Fig 6E and 6F). Thus, the analysis of electron micrographs showed that nimesulide caused important ultrastructural changes on *T*. *cruzi* epimastigotes.

## Discussion

The search for new molecules that have effective and safe activity against *T*. *cruzi* has motivated a series of studies in several research groups. Chagas disease is a public health problem [1] and the therapeutic action of the two drugs available to treat infected patients are benznidazole and nifurtimox which has severe limitations mainly in the chronic phase of the infection [28]. Several compounds were tested in order to identify their activity, including those from the class of nitroaromatics [29]. Indeed, interest in molecules containing the nitro group has undergone a resurgence in recent years, due to a greater understanding of the role of nitro-reductases involved in their activation and also on the mechanisms by which parasites develop resistance [30]. Our goal was to evaluate the ability of the nitroaromatic drug nimesulide to inhibit the growth of the different evolutive forms of *T*. *cruzi*, in an attempt to propose the repositioning of this drug for the treatment of Chagas disease. The process of discovering other therapeutic purposes for commercialized drugs brings significant savings in time and costs [5]. This approach is particularly attractive when applied to neglected diseases, since there are no significant investments in the development of new drugs in this field [31]. Our results pointed to the trypanocidal activity of nimesulide on *T*. *cruzi* epimastigotes. Interestingly, the trypanocidal activity shown by nimesulide disappeared after the chemoselective reduction of the nitro group present in its molecule, that stimulated us to study the role of the nitro group as the possible pharmacophore for the trypanocidal activity observed to nimesulide. In addition, tests carried out against the other evolutive forms of the parasite revealed that nimesulide inhibited both the replication of amastigotes and the release of trypomastigote forms when comparing to benznidazole. In addition, the results of flow cytometry tests indicated that after 24 hours of treatment nimesulide shows toxic effects on *T*. *cruzi* like benznidazole. The positive marking for both probes (Annexin-V/PI staining) used to distinguish the different ways of cell death showed that nimesulide induced two simultaneous mechanisms of parasite death by necrosis and apoptosis. These results are in complete agreement to those shown by Tay and co-workers [32]. In this study, the group showed the effects of nimesulide on isolated mouse liver mitochondria which experienced both uncoupling as calcium-dependent membrane permeability transition (MPT). These events led to the increase in mitochondrial oxidative stress. Another important result highlighted by the authors shows that the corresponding amine (obtained by chemoselective reduction of nitro group of nimesulide) does not provide the same mitochondrial effects. In fact, the authors describe a protective effect promoted by amine against ROS production [32]. These results corroborate our findings on the effects of both compounds (nimesulide and its corresponding amine) on *T*. *cruzi* epimastigote cells. It is well known in the literature that this kind of deleterious effects in mitochondria induces cell death by mixed mechanisms, that is both by necrosis and apoptosis [33–35].

The ultrastructural organization of the different evolutive forms of *T*. *cruzi* has been extensively studied by the transmission electron microscopy (TEM) technique [36]. In **Figs** 5A and 5C and 6A and 6D we can see the main organelles found in the complete epimastigote form of the parasite used as a control in our work which are in full agreement with the data previously described, in the anterior region of the parasite [36–38]. Epimastigotes of *T*. *cruzi* have a spherical nucleus and nuclear envelope with pores, and a nucleolus in the central portion. The flagellum protrudes from the half of the protozoan’s body. The kinetoplast, a specific region of the mitochondria in which the DNA is concentrated, has a disk shape [15, 36, 37]. The shape of the kinetoplast and its position in relation to the nucleus are characteristic in the different evolutive forms of *T*. *cruzi*, and in epimastigotes it is found between the flagellar pocket and the nucleus, in the anterior region of the parasite [36, 37].

Herein the TEM analysis of epimastigotes maintained in axenic culture revealed the integrity of the parasite, when compared to the literature [15, 36]. All the characteristics could be observed in the obtained micrographs, as shown in **Figs** 5A and 5C and 6A and 6D. In turn, *T*. *cruzi* epimastigotes treated with nimesulide demonstrated important ultrastructural changes, mainly in the loss of mitochondrial membrane morphology and strong kinetoplast compaction. Mitochondrial changes are common in both necrosis and apoptosis, and in the case of necrosis, a concomitant increase in cytoplasmic vacuolization is observed [17]. The increase in cytoplasmic vacuolization was also observed in our study. Data from literature demonstrate that reservosomes play an important role in the integrity of *T*. *cruzi* epimastigotes affecting parasite’s lipidic metabolism which is impaired or may even start a proteolytic process, resulting in cell death [17]. In the present study, we observed that the treatment of epimastigotes with nimesulide caused a clear disorganization of epimastigote’s reservosomes, pointing to an imbalance in the parasite’s metabolism. Another ultrastructural aspect found in our study was the abnormal pattern of heterochromatin distribution in the treated epimastigotes indicating the damage caused by nimesulide in this organelle in a process suggestive of apoptosis [18]. Thus, we were led to believe that the ultrastructural effects observed after treatment with nimesulide point to cell death due both to apoptosis and necrosis.

An important and advantageous aspect to be considered about the repositioning of nimesulide for the treatment of Chagas disease patients is that inflammatory processes are among the most harmful effects caused by parasites in the chronic phase of the infection. Persistent cardiac tissue parasitism causes serious inflammatory complications, causing irreversible myocardial damage [38]. In this sense, the use of an anti-inflammatory drug can be advantageous to alleviate the inflammatory damage caused to patients in the chronic phase of the disease.

In general, the different methodological approaches, involving repositioning, consider as an important aspect the fact that the effectiveness of the candidate drug is associate with the minimization of side effects to the patient. In addition, many studies compare the results observed in monotherapy with combination with another drug. For example, Guedes-da-Silva and co-workers [39] investigated the effectiveness of oral doses of the experimental 14α-demethylase inhibitor, VNF, both in monotherapy as in combination with BNZ in infected mouse. All tested mice groups resulted in >99.9% of parasitemia decrease and 100% animal survival. In another recent study, the antibiotic Clofazimine and the antihypertensive Benidipine were evaluated by in combination with low doses of Benznidazole against the intracellular amastigotes of *T*. *cruzi* both *in vitro* and in a murine model of chronic infection with promising results [40]. In present in vitro study, we tested nimesulide against *Trypanosoma cruzi* and showed its toxic action on different evolutive forms of the parasite. The results obtained herein open a perspective to a future study on the effects of the association of nimesulide and benznidazole, both *in vitro* as *in vivo*. Another point to be highlighted is the existence of *Trypanosome cruzi* strains resistant to benznidazole. Although we observed nimesulide shows IC_50_ values higher than benznidazole, the perspective of the antiparasitic action of this NSAID in benznidazole resistant strains may bring benefits to the chagasic patients enabling a more effective treatment. Furthermore, the treatment with the association of the two drugs in reduced doses could be shorter, more effective, and safer. A similar study was carried out by Torrico et al. [41] who found that the combination of benznidazole and fosravuconazole reduced the treatment time, which improved patient compliance due to the minor side effects produced. Further studies regarding the safety of using nimesulide in the treatment of chagasic patients should consider that the most important adverse effects observed for nimesulide, and also for other nitroaromatic drugs, involve liver damage. However, at the recommended therapeutic doses, these effects are associated with issues of idiosyncrasies and individual predispositions [42]. Furthermore, many of the problems are associated with the use of the drug without professional supervision and with its easy acquisition without a prescription. Finally, it should be noted that pharmacokinetic studies available for nimesulide indicate the drug reaches an average plasma concentration of 39.15 μM, in a period of 3.19 h [43], when administered in an oral dose of 200 mg, which is a safe dose when using nimesulide as anti-inflammatory drug. Although the high affinity of nimesulide for serum proteins, mainly albumin, is well described in the literature [44], the plasma concentrations of nimesulide described by Kim and co-workers [43] are close, and even higher, to the effective trypanocidal concentrations of nimesulide assayed in this work.

In conclusion, the set of results obtained in this work shows the potential of nimesulide to be repositioned as an anti-Chagas drug or yet to serve as a starting point for the design of a new class of antichagasic analogs. The results obtained herein shows that nimesulide drug has remarkable effects on the different evolutive forms of *T*. *cruzi*. It was demonstrated that the antiparasitic effects of the drug are related to the nitro group present in its structure, since the aniline derivative, obtained by chemoselective reduction of nimesulide was not active. The data obtained in this study, when compared to those previously described in the literature, suggest the interference of nimesulide in the redox balance of the parasite’s cell, promoting its death mainly by oxidative stress. It was found that epimastigotes treated with nimesulide follow mixed mechanisms of death, due both to apoptosis and necrosis, which correlates with the ultrastructural changes observed.

## Supporting information

S1 Text Analytical equipment used in the characterizations and determination of purity grades of compounds. (DOCX)

S1 Fig NMR ^1^H spectrum of nimesulide in CDCl_3_ at 500 MHz. (DOCX)

S2 Fig ^13^C NMR spectrum of nimesulide in CDCl_3_ at 125MHz. (DOCX)

S3 Fig Mass spectrum of nimesulide. (DOCX)

S4 Fig RP-HPLC of nimesulide. (DOCX)

S5 Fig NMR ^1^H spectrum of reduced nimesulide in CDCl_3_ at 500 MHz. (DOCX)

S6 Fig ^13^C NMR spectrum of reduced nimesulide in CDCl_3_ at 125 MHz. (DOCX)

S7 Fig Mass spectrum of reduced nimesulide. (DOCX)

S8 Fig RP-HPLC of reduced nimesulide. (DOCX)

S1 Table ^1^H NMR and ^13^C NMR data of nimesulide. (DOCX)

S2 Table ^1^H and ^13^C NMR data of reduced nimesulide. (DOCX)
