# Supplementary material for: Drug repurposing for Chagas disease: In vitro assessment of nimesulide against Trypanosoma cruzi and insights on its mechanisms of action
Source: PLoS One. 2021 Oct 22;16(10):e0258292. doi: 10.1371/journal.pone.0258292 (PMC8535186; doi:10.1371/journal.pone.0258292)
Supplement: S1 Text — (DOCX) [file pone.0258292.s001.docx]

**S1 Text. Analytical equipment used in the characterizations and determination of purity grades of compounds***.* The ^1^H-NMR (500 MHz) and ^13^C-NMR (125 MHz) spectra were recorded on a Bruker Ultrashield Plus Spectrometer (BrukerBioSpin GmbH, Rheinstetten, Germany) operating at 500 MHz for ^1^H and 125 MHz for ^13^C. ^1^H and ^13^C-NMR shifts (δ) are reported in parts *per* million with respect to CDCl_3_ (*δ* 7.29 ppm for ^1^H; and *δ* 77.0 ppm for ^13^C) and coupling constants (*J*) in Hertz [Hz]. The low-resolution mass spectra were carried out on a Shimadzu GCMS-QP2010 Plus (Shimadzu Inc., Kyoto, Japan). Analytical conditions: Column: VF-5MS, 30 m × 0.25 mm × 0.25 µm (Varian Inc., Santa Clara, CA, USA); Column temperature: 200 ^o^C for one minute and then increasing to 290 ^o^C at a rate of 10 ^o^C min^-1^ and holding for 40 minutes; Injector temperature: 543 K. Reversed phase high performance liquid chromatography (RP-HPLC) were performed on a Shimadzu chromatograph consisting of two LC-20AT series pumps; SPD-M20A series diode array detector; and Rheodyne 7125i injector with 20 *μ*L loop. Equipment control and data acquisition was done using the LCSolution software (Shimadzu, Kioto, Japan). The analyzes were performed in a C-18 reverse phase analytical column of 150 x 4.6 mm, 5 *µ*m of particle (Allure Restek, PA, USA), maintained at 30ºC. The mobile phase used was a mixture of acetonitrile (98%, solvent B) and water (2%, solvent A). The volume of injection was 20 *μ*L and the separation was performed in isocratic mode (constant flow of 1.2 mL.min^-1^).
