# Supplementary material for: Drug repurposing for Chagas disease: In vitro assessment of nimesulide against Trypanosoma cruzi and insights on its mechanisms of action
Source: PLoS One. 2021 Oct 22;16(10):e0258292. doi: 10.1371/journal.pone.0258292 (PMC8535186; doi:10.1371/journal.pone.0258292)
Supplement: S5 Fig — (DOCX) [file pone.0258292.s006.docx]

S5 Fig. ^13^C NMR spectrum of reduced nimesulide in CDCl_3_ at 125 MHz.
