# Supplementary material for: Drug repurposing for Chagas disease: In vitro assessment of nimesulide against Trypanosoma cruzi and insights on its mechanisms of action
Source: PLoS One. 2021 Oct 22;16(10):e0258292. doi: 10.1371/journal.pone.0258292 (PMC8535186; doi:10.1371/journal.pone.0258292)
Supplement: S1 Table — (DOCX) [file pone.0258292.s010.docx]

**S1 Table. ^1^H NMR and ^13^C NMR data of nimesulide.**





| Position |  | | δ ^1^H (ppm) |  | δ^13^C (ppm) |
| --- | --- | --- | --- | --- | --- |
| 1 | | --- | | | 146.37 (C) |
| 2 | | --- | | | 143.72 (C) |
| 3 | | 7.80-7.78 (d, 1H, *J*=8.0 Hz) | | | 125.95 (CH) |
| 4 | | --- | | | 154.17 (C) |
| 5 | | 8.04-8.01 (d, 1H, *J*=8.0 Hz) | | | 130.69 (CH) |
| 6 | | 7.68 (d, 1H, *J*=8.0 Hz) | | | 130.69 (CH) |
| CH_3_ | | 3.19 (s, 3H) | | |  |
| 1’ | | 7,11-7,09 (m, 1H) | | | 119.41 (CH) |
| 2’ | | 7.49-7.47 (m, 1H) | | | 119.69 (CH) |
| 3’ | | 7.32 (m, 1H) | | | 111.91 (CH) |
| 4’ | | 7.49-7.47 (m, 1H) | | | 119.69 (CH) |
| 5’ | | 7.11-7.09 (m, 1H) | | | 117.38 (CH) |
| 6’ | | --- | | | 133.97 (C) |
