# Supplementary material for: Drug repurposing for Chagas disease: In vitro assessment of nimesulide against Trypanosoma cruzi and insights on its mechanisms of action
Source: PLoS One. 2021 Oct 22;16(10):e0258292. doi: 10.1371/journal.pone.0258292 (PMC8535186; doi:10.1371/journal.pone.0258292)
Supplement: S2 Table — (DOCX) [file pone.0258292.s011.docx]

**S2 Table. ^1^H and ^13^C NMR data of reduced nimesulide.**





| Position |  | | δ ^1^H (ppm) |  | | δ^13^C (ppm) |
| --- | --- | --- | --- | --- | --- | --- |
| 1 | | --- | | | 115.25 (C) | |
| 2 | | --- | | | 149.59 (C) | |
| 3 | | 6.31-6.28 (d, 1H, *J*=8.0 Hz) | | | 103.25 (CH) | |
| 4 | | --- | | | 156.67 (C) | |
| 5 | | 6.05 (d, 1H, *J*=8.0 Hz) | | | 109.29 (CH) | |
| 6 | | 7.01-6.99 (d, 1H, *J*=8.0 Hz) | | | 124.03 (CH) | |
| SO_2_CH_3_ | | 2.88 (s, 3H) | | | 40.46 (CH_3_) | |
| 1’ | | 7.18-7.15 (m, 1H) | | | 119.78 (CH) | |
| 2’ | | 7.44-7.40 (m, 1H) | | | 131.00 (C) | |
| 3’ | | 7.07-7.05 (d, 1H) | | | 130.38 (CH) | |
| 4’ | | 7.44-7.40 (m, 1H) | | | 131.00 (C) | |
| 5’ | | 7.18-7.15 (m, 1H) | | | 119.78 (CH) | |
| 6’ | | --- | | | 153.65 (C) | |
| NH | | 8.79 (s, 1H) | | | --- | |
| NH_2_ | | 5.28 (s, 2H) | | | --- | |
